# Psychological Flexibility in Depression Relapse Prevention: Processes of Change and Positive Mental Health in Group-Based ACT for Residual Symptoms

**DOI:** 10.3389/fpsyg.2020.00528

**Published:** 2020-03-27

**Authors:** Tom Østergaard, Tobias Lundgren, Robert D. Zettle, Nils Inge Landrø, Vegard Øksendal Haaland

**Affiliations:** ^1^Department of Psychiatry, Sørlandet Hospital, Arendal, Norway; ^2^Clinical Neuroscience Research Group, Department of Psychology, University of Oslo, Oslo, Norway; ^3^Center for Psychiatry Research, Department of Clinical Neuroscience, Karolinska Institutet, Stockholm, Sweden; ^4^Department of Psychology, Wichita State University, Wichita, KS, United States; ^5^Division of Psychiatry, Diakonhjemmet Hospital, Oslo, Norway

**Keywords:** acceptance and commitment therapy, psychological flexibility, multilevel mediation, depression, positive mental health

## Abstract

Relapse rates following a depressive episode are high, with limited treatments available aimed at reducing such risk. Acceptance and commitment therapy (ACT) is a cognitive-behavioral approach that has gained increased empirical support in treatment of depression, and thus represents an alternative in relapse prevention. Psychological flexibility (PF) plays an important role in mental health according to the model on which ACT is based. This study aimed to investigate the role of PF and its subprocesses in reducing residual symptoms of depression and in improving positive mental health following an 8-week group-based ACT treatment. Adult participants (75.7% female) with a history of depression, but currently exhibiting residual symptoms (*N* = 106) completed measures before and after intervention, and at 6 and 12-month follow-up. A growth curve model showed that positive mental health increased over 12-months. Multilevel mediation modeling revealed that PF significantly mediated these changes as well as the reduction of depressive symptoms, and that processes of acceptance, cognitive defusion, values and committed action, in turn, mediated increased PF.

## Introduction

According to the World Health Organization (WHO), major depressive disorder (MDD) represents one of the greatest global health challenges (WHO, 2017). Relapse rates after MDD are high, with an estimated 75% experiencing more than one episode (Boland and Keller, 2009). Previously depressed persons will often experience depressive symptoms between episodes at different levels of severity (Judd et al., 1998; Möller, 2008; Nil et al., 2016), that are predictive of recurrence of MDD (Judd et al., 1998; Paykel, 2008), suggesting that interventions that target them could be effective in reducing relapse. Residual symptoms of depression (i.e., subclinical symptoms such as lingering dysphoria) have also been associated with impaired psychosocial functioning, fatigue, and decreased ability to work (Kennedy et al., 2007; Trivedi et al., 2009; Fava et al., 2014). A large amount of the burden associated with depression could be averted by interventions that prevent relapse or recurrence (Vos et al., 2004).

Recent mindfulness and acceptance-based cognitive-behavioral interventions, such as mindfulness-based cognitive therapy (MBCT; Segal et al., 2002) and acceptance and commitment therapy (ACT; Hayes et al., 2012b), have gained increased attention as treatments for depression. MBCT, in particular, which was specifically designed to be a depression relapse-prevention intervention, has shown promise in doing so (Teasdale et al., 2000; Ma and Teasdale, 2004).

Acceptance and commitment therapy shares some conceptual similarities with MBCT, but differs on philosophical, methodological, and strategic dimensions (Zettle and Gird, 2017). ACT combines acceptance and mindfulness processes with commitment and behavior-change processes within a transdiagnostic model that focuses on pathogenic processes hypothesized to be common in different forms of human suffering (Zettle, 2007). ACT has been found to be an efficacious treatment for depression when evaluated in individual, self-help, and group formats (Forman et al., 2007; Fledderus et al., 2012; Folke et al., 2012; A-Tjak et al., 2018; Kyllönen et al., 2018). An important aspect of ACT is that it expands the concept of psychopathology by focusing on how to adaptively respond to both subclinical and clinical levels of suffering in ways that empower individuals to act in accordance with their needs and values (Hayes, 2012). Rather than just focusing on symptomatic relief, ACT explicitly seeks to promote mental health and well-being by increasing meaningfulness and valued living (Hayes et al., 2012a).

World Health Organization (WHO) defines mental health as “…a state of well-being in which the individual realizes his or her own abilities, can cope with the normal stresses of life, can work productively and fruitfully, and is able to make a contribution to his or her community” (WHO, 2004, p. 10). The notion of mental health and psychopathology as two separate, but correlated dimensions of psychological functioning has gained increased support (Greenspoon and Saklofske, 2001; Keyes, 2005; Kashdan and Rottenberg, 2010). A decrease or absence of psychopathology doesn’t necessarily indicate thriving mental health (Keyes, 2005). Ideally, an intervention should target both dimensions, decreasing psychopathology and improving psychological well-being, in order to enhance the ability to handle difficult aspects of life.

Acceptance and commitment therapy regards the concept of psychological flexibility (PF) in being able to make necessary behavioral adjustments in the pursuit of valued ends (Hayes et al., 2006) as essential in this two-fold undertaking. PF has been related to a number of positive benefits such as self-regulation (Bonanno et al., 2004), self-determination (Deci and Ryan, 2000), stress-coping (Cheng, 2001), goal-attainment (Tamir, 2009), and social functioning (Gross and John, 2003). The opposite of PF, psychological *in*flexibility, has been associated with processes that are central in depression (Zettle, 2007; Kashdan and Rottenberg, 2010). An example of *in*flexibility is ruminative brooding over negative emotional states and related thoughts, which particularly has been shown to play an important role in the initiation, maintenance, and recurrence of depression (Nolen-Hoeksema et al., 2008).

Depression from an ACT perspective is seen as a consequence of psychological *in*flexibility, where struggle with inner content (e.g., thoughts, feelings, and bodily experiences) leads to avoidant and rigid behavior that limits individuals from taking steps that would improve their quality of life and emotional well-being. In order to promote PF, the model of human functioning on which ACT is based suggests that six interdependent processes are central: (a) acceptance, (b) cognitive defusion, (c) contact with the present moment, (d) self-as-context, (e) values, and (f) committed action (Hayes et al., 2011).

*Acceptance* involves meeting inner experiences (such as thoughts, emotions, bodily sensations, and memories), both pleasant and unpleasant, from moment-to-moment with non-judgmental awareness (Hayes et al., 2012b). Acceptance is not an attitude or feeling, but rather an action taken toward inner experiences (Twohig and Levin, 2017). Being more open and attentive to inner experiences increases the ability to choose value-based behaviors. Acceptance is an alternative to experiential avoidance, which is an unwillingness to be in contact with inner experiences that manifests itself through behavioral strategies aiming to reduce or avoid them. Experiential avoidance has been identified as an important factor contributing to depression (Cribb et al., 2006; Shallcross et al., 2010; Rueda and Valls, 2016) and commonly takes the form of rumination, where deliberate efforts are made to think through it (Zettle, 2007).

*Cognitive defusion* represents a way of responding to thoughts, reasons, and life stories that entails disengaging from them (Zettle, 2007). An opposing process of fusion occurs when such cognitive content dominates behavior by being held as literally true (Gillanders et al., 2014). Cognitive fusion has been associated with different forms of psychological distress (Ferreira et al., 2014; Fergus, 2015; Solé et al., 2015; Hapenny and Fergus, 2017), and several studies have found a relationship between cognitive fusion and depression (e.g., Zettle et al., 2011; Gillanders et al., 2014; Dinis et al., 2015; Bardeen and Fergus, 2016).

*Contact with the present moment* is the ability to take a step back from being caught up in the past or a constructed future, and direct attention to the here-and-now in a non-judgmental way (Zettle, 2007). This process is also referred to as mindfulness, although traditions within mindfulness may differ somewhat on what is identified as processes of change (Hayes et al., 2012b). ACT uses different mindfulness exercises to promote attentional flexibility and enhance skills that facilitate other processes. Awareness directed toward sensory and physical experiences taking place from moment-to-moment may prevent being stuck in verbally constructed judgments, evaluations, and comparisons (Luoma and Villatte, 2012). As such, mindfulness represents an alternative to rumination, and facilitates more flexible and meaningful responses to both internal and external challenges. Mediating effects of mindfulness on outcomes of depression have been found in several studies (e.g., Ramel et al., 2004; Kuyken et al., 2010; Pots et al., 2016).

*Self-as-context* refers to awareness and self-perspective from which one creates distance from conceptualized identities, like for instance “I’m broken” or “I’m depressed.” Conceptualized views of the self constrict behavioral actions that could provide depressed individuals with new experiences and insight. Overidentification with a certain life narrative may instead help perpetuate a self-fulfilling prophecy about the future. For some time, there has been a relative lack of studies of this process because of difficulties in measuring it. However, the recent development of assessment instruments has produced preliminary findings suggesting a supportive role of self-as-context in enhanced mental health (Yu et al., 2016, 2017; Moran et al., 2018; Zettle et al., 2018).

*Values* represent life areas and qualities that are important to the individual, and that can be purposely used to guide behavior. A loss of meaning has been identified as a central component in the development of depression (Lewis, 1995; Hayes et al., 2012b; Ratcliffe, 2012; Frankl, 2014). From an ACT point of view, contact with meaning often is lost because of internal struggles with difficult emotions, resulting in a detachment from valued domains of living (Hayes et al., 2012b). In this way, depression becomes a secondary emotion that emerges because of difficulties in accepting normal and adaptive emotional reactions to distressing life events (Zettle, 2015). Various studies have found a relationship between values and psychological distress (Wilson et al., 2010; Vilardaga et al., 2011; Vowles et al., 2011).

*Committed action* represents behavior that brings specified values to life. Concrete goals are an important part of developing short and long term behavior changes and serve as a link between values and action. The primary task in ACT is not to target inactivity broadly defined, but to foster value-based activities more specifically (Zettle, 2007).

To understand the mechanisms through which ACT works, investigating PF, its processes, and their relationship to therapeutic outcome is necessary (Kazdin, 2007). A recent meta-analysis focusing on clinical studies found PF to mediate improvement in different aspects of mental health (Stockton et al., 2019). They found mediational evidence for the process of acceptance to be sound and thoroughly documented. Cognitive defusion and committed action also were found to be robust mechanisms of change, although with substantially fewer studies that suggested they were processes unique to ACT. The other processes within the PF model showed more sparse and inconsistent evidence.

Several studies have found evidence of an association between increased PF and symptomatic relief (e.g., Forman et al., 2007; Jeffcoat and Hayes, 2012; Fledderus et al., 2013; Gaudiano et al., 2013; Walser et al., 2013; Dalrymple et al., 2014). For instance, Fledderus et al. (2013) found that improved PF mediated the effect of an ACT intervention targeting mild to moderate depression and anxiety. However, several of these studies were limited by small samples, short follow-ups, and a reliance on correlational evidence. A number studies have also provided evidence of a relation between PF and psychological well-being (e.g., Bond and Bunce, 2003; Kashdan et al., 2006; Fledderus et al., 2010; Wersebe et al., 2018), suggesting importance of forwarding PF to enhance mental health.

Research on processes of ACT has also often been conducted with non-clinical or subclinical samples in laboratory-based studies where it is possible to control and manipulate variables to test theoretical hypotheses. A meta-analysis of 66 laboratory-based process studies found significant positive effect sizes (ESs) for acceptance, cognitive defusion, present moment awareness and values compared to control conditions (Levin et al., 2012).

A broad exploration of the processes in ACT is needed to determine and refine which ones are central for change to occur (Hofmann and Hayes, 2019). There is a particular lack of clinical studies with long follow-ups that have examined the relative contributions of PF subprocesses as putative mechanisms of change in ACT (Stockton et al., 2019). Research responsive to this omission may have both conceptual as well as clinical practice implications. Increased PF as a broad-band mediator of therapeutic change has been reasonably established. Less, however, is known about whether the relative contributions of the subprocesses, such as cognitive defusion and mindfulness, that ostensibly in turn mediate increased PF vary as a function of the form or type of human suffering being addressed. This leads to the possibility that the impact of ACT might be optimized by matching up different therapeutic components with the subprocesss of PF they target, at least based in part on diagnostic considerations. For example, procedures that mediate increased valued action may be more critical in successfully alleviating depression than substance abuse.

**FIGURE 1:**
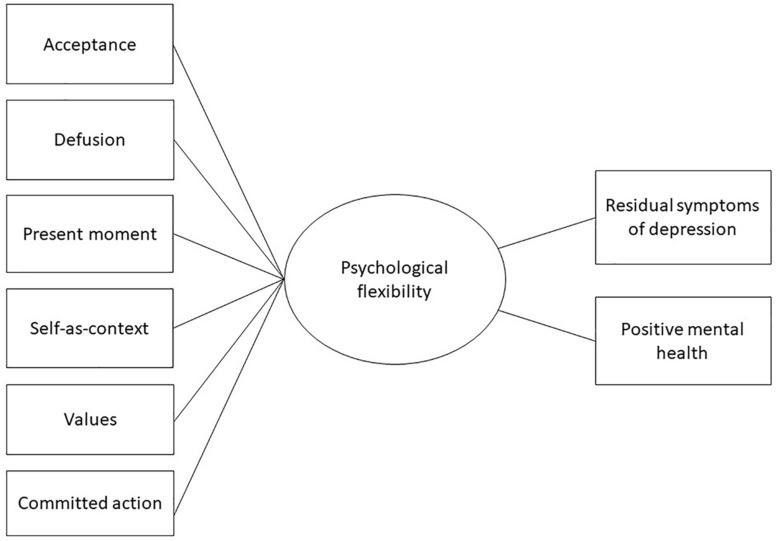
Illustration of the subprocesses, their relationship to PF, and effect of PF on residual symptoms and positive mental health.

Figure 1 displays a model of the subprocesses, their relationship to PF, and effect of PF on residual symptoms and positive mental health. PF has been identified as central in meaningful behavior change, but to our knowledge a mediational evaluation of the components of PF in relation to it has not previously been undertaken. Our primary purpose was first to investigate the mediating effect of PF on residual symptoms of depression and positive mental health outcomes of an 8-week group-based ACT intervention. Furthermore, we subsequently also explored which related subprocesses contributed to PF as a superordinate mechanism of therapeutic change. Finally, this study also provided an opportunity to further examine improvement in positive mental health outcomes in relationship to reductions in residual symptoms of depression. This leads to the following research questions:

Is there a mediating effect of psychological flexibility on residual symptoms of depression and positive mental health outcomes in group-based ACT over 12-months follow-up?

Which subprocesses mediated the superordinate process of psychological flexibility during a year follow-up?

What effect does group-based ACT have on positive mental health, and what is the relationship to reductions in residual symptoms of depression?

## Materials and Methods

### Design

Participants in the present study also took part in a larger trial that explored the effect of group-based ACT in reducing residual symptoms of depression and also investigated the possible benefits of first receiving a 2-week experimental attentional bias modification (ABM) procedure (Østergaard et al., 2019). This parent study consisted of two phases. In phase 1, 244 participants from Oslo or Sørlandet (southern Norway) were randomized to receive ABM (a computerized attention training procedure) or control condition. In phase 2, a quasi-experimental design was introduced and only participants from Sørlandet received group-based ACT. ACT significantly reduced residual symptoms compared to a control condition (no specific intervention apart from regular health care provided by Norwegian health services) and over 12-months post-treatment, both self-reported and clinician-rated measures continued to improve with large ESs. However, contrary to expectations, ABM did not augment the benefits of ACT.

The present study only included participants from Sørlandet who received ACT and completed both outcome and process measures at all follow-ups, except at 1-month (due to limiting patient burden), making it possible to investigate potential mediational effects as the focus of this paper. Participants from Oslo were not administered the same process measures and were therefore not included. Because there were no differences in outcome between those who had received ABM before the group and those who did not, we combined the two into one sample of 106 participants.

The parent study was approved by the Norwegian Regional Committee for Medical and Health Research Ethics, reference number 2014/1989, with its period of recruitment and follow-up extending from May 2015 to October 2018.

### Participants

In order to participate in the study the participants had to be between 18 and 65 years old and in remission with a history of MDD as established by the Mini International Neuropsychiatric Interview, version 6.0.0. (MINI) (Lecrubier and Sheehan, 1997). Exclusion criteria were current or past neurological illness, bipolar disorder, psychosis, drug addiction, and attention deficit disorder with and without hyperactivity (ADHD and ADD).

The participants were on average 41 years old, with the majority being women (75.7%) of higher education (64.4%). All had a history of depression and experienced residual symptoms of depression. Sample characteristics are presented in Table 1.

**TABLE 1:** Sample characteristics, given as number and proportion for categorical characteristics and as mean and standard deviation for quantitative characteristics.

Characteristics	Group-Based ACT *N* = 106
**Gender**	
Males	26 (24.5)
Females	80 (75.5)
Age	40.77 (11.9)
**Education**	
Lower than university	37 (34.9)
University or higher	67 (63.2)
Missing	2 (1.9)
**Comorbidity**	
Yes	55 (51.9)
No	51 (48.1)
Number of depression episodes before treatment	6.18 (6.7)
Missing	1 (0.9)

### Procedure

Participants were recruited from specialist mental health care centers, regular general practitioners, and via self-referrals. A clinical assessment was performed including a study-specific questionnaire (where demographic information, including medication status, and treatment history were obtained), and administration of the MINI (Lecrubier and Sheehan, 1997). The evaluations were conducted by psychologists or psychology students who had received training and supervision in the assessment package.

According to the *Clinical Trials* protocol current MDD was an exclusion criterion. However, 15 participants with ongoing depression were included in error. Consistent with an intention-to-treat procedure all data were included. Because excluding data from these participants did not affect the results, they were included in all reported analyses. Tables presenting these analyses can be found in Supplementary Tables S4, S5.

Group-based ACT was administered by duos of eight experienced psychologists (three woman and five men) who had been trained in the approach and received supervision. They were instructed to follow a manual (in Norwegian), which had been developed for this study, specifying treatment ingredients, intervention structure and therapist behavior. ACT consisted of 8 weekly sessions with 6–10 participants per group. The treatment aimed to promote and strengthen PF, and combined psychoeducation with experiential exercises (e.g., getting in contact with a certain process through an illustrative activity) and group processing of both elements. Mindfulness exercises were conducted during each session to increase present moment awareness and facilitate other ACT processes. See Østergaard et al. (2018) for a more detailed description of the protocol.

All measures were administered and completed at baseline, 2, 6, and 12-months. Except for Hamilton Rating Scale, all measures were completed by participants at home the day before the follow-up.

### Diagnostic Measure

The MINI-International Neuropsychiatric Interview (MINI) (Lecrubier and Sheehan, 1997) is a structured interview compatible with DSM-IV and ICD-10 criteria of determining psychiatric diagnoses. The MINI has shown has been found to be a psychometrically sound of depression in both psychiatric and primary care (Otsubo et al., 2005; de Azevedo Marques and Zuardi, 2008; Pettersson et al., 2018).

### Symptomatic Measures

Beck Depression Inventory-II (BDI-II) (Beck et al., 1996) is a psychometrically sound measure of depressive severity consisting of 21 items. The Norwegian translation of the BDI-II displays high internal consistency (α = 0.92 at baseline in this study), and acceptable levels of convergent and discriminative validity (Aasen, 2001).

Hamilton Rating Scale for Depression (HRSD) (Hamilton, 1960, 1967) is a widely used semi-structured, clinical interview measuring the severity of a range of 17 affective, behavioral, and biological symptoms of depression. The HRSD has acceptable psychometric properties (Rabkin and Klein, 1987) with good internal consistency (α = 0.91) and a high correlation (*r* = 0.62) with the BDI-II in this study at baseline.

Mental Health Continuum – Short Form (MHC-SF) (Keyes, 2002, 2009) measures positive mental health with 14 items that assess the degree of well-being in the past month. MHC-SF has been found to have good psychometric properties (Lamers et al., 2011) with higher scores reflective of positive mental health. It has previously been translated into Norwegian by Langeland et al. (2013) and showed good internal consistency at baseline (α = 0.93).

### Process Measures

Acceptance and Action Questionnaire-II (AAQ-II) (Bond et al., 2011) is a seven-item measure of PF rated on a Likert scale (7 = *always*; 1 = *never true*), with higher totals, as scored in this study, indicating less PF. AAQ-II has previously been found to be unidimensional and acceptably reliable and valid. A recent investigation of the psychometric properties of the Norwegian translated version of the AAQ-II (Østergaard et al., 2020) found satisfactory levels of internal, concurrent, and convergent validity. The AAQ-II showed good internal consistency at baseline (α = 0.86) in this study.

Cognitive Fusion Questionnaire (CFQ) (Gillanders et al., 2014) is a seven-item questionnaire that on a seven-point Likert scale assesses cognitive fusion. The CFQ displayed good psychometric properties based on preliminary findings (Gillanders et al., 2014). The CFQ was forward and back-translated into Norwegian with independent checking for the use in the present study. The CFQ showed an acceptable level of internal consistency at baseline (α = 0.94) in this study.

Bull’s Eye Value Survey (BEVS) (Lundgren et al., 2012) evaluates value-congruent behavior in four domains: (a) health, (b) leisure activities, (c) family, and (d) work/education. Separate marks are placed on a picture of a dartboard indicating the degree to which respondents are behaving in ways consistent with each domain. Placement of marks closer to the bull’s eye results in higher scores reflecting greater levels of self-defined value attainment. The BEVS has shown good temporal stability and has many properties supporting its construct validity (Lundgren et al., 2012). It was translated into Norwegian from Swedish with the assistance of its creator (Lundgren et al., 2012). It displayed an acceptable level of internal consistency at baseline (α = 0.69).

Engaged Living Scale (ELS) (Trompetter et al., 2013) was developed to provide a broad assessment of how valued life activities are pursued, incorporating predefined statements of values. By contrast, values, as assessed by the BEVS, are self-defined. Thus, these two instruments appear to reflect somewhat different, albeit related facets of values and committed action.

The ELS consists of 16 questions scored on a five-point Likert scale with higher scores indicating a higher degree of engaged living. A study by Trompetter et al. (2013) found ELS to be a valid and reliable measure of an engaged response style. The ELS was forward and back-translated into Norwegian with independent checking for use in the present study. In the present study, internal consistency at baseline was good (α = 0.92).

Philadelphia Mindfulness Scale (PHLMS) (Cardaciotto et al., 2008) is a psychometrically sound, 20-item questionnaire that assesses present-moment awareness and acceptance as two key components of mindfulness. The PHLMS was forward and back-translated into Norwegian with independent checking for use in the present study. The awareness and acceptance component scales exhibited high levels of internal consistency at baseline with respective alpha coefficients of 0.84 and 0.91.

### Statistical Analyses

The aims of this study were twofold. First, we examined if changes in PF over time as a superordinate process as measured by the AAQ-II mediated decreases in residual symptoms of depression and improvement in mental health. Second, we explored if changes over time in specified subprocess measures mediated improved PF. Because the data are hierarchical (Bauer et al., 2006), a multilevel modeling (MLM) approach with two-levels was employed, where repeated measures (level 1) were nested within participants (level 2) (Curran and Bauer, 2011). Similar to simple mediation, multilevel mediation with multiple measurements (commonly known as lower-level mediation) investigates the total effect (*c)* of the independent variable (time) on the outcome variable to determine if a process variable mediates the relationship. The MLM encompasses the effect of time on the process variable (path *a*), the effect of changes in the process variable following changes in the outcome variable when time is held constant (path *b*), the direct effect of time on the outcome variable when controlling for effect of mediator (path *c*′), the indirect effect of time on the outcome variable represented by changes in the process variable (*ab*). A multilevel mediation model analyzes the pathways involving time by including the effects of outcome variables both within each participant and across the sample. An advantage of MLM is that all available data are included, and correlations between the repeated measures are controlled for (Kenny et al., 2003).

**FIGURE 2:**
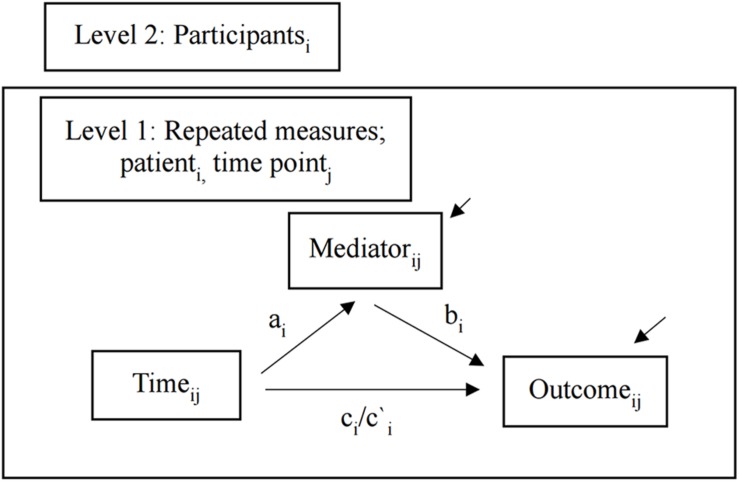
Multilevel mediation model. Lower level mediation model with two levels as estimated in the present study. Subscripts specify that measures vary across patients *i* and time *j*. Arrows between variables and path labels indicate hypothesized causal effects. Residuals are indicated by raised arrows

We applied the following mediation model: (a) time (months) as the independent variable, (b) “process variables at month j” as potential mediators, and (c) “outcome variable at month j” as the dependent variable. Multilevel mediation analysis was conducted in PASW 25.0 (IBM) using the “MLmed” macro by Rockwood and Hayes (2017) on an intention-to-treat basis. Different independent multilevel analyses were conducted where potential mediators were estimated individually. Only within-group effects were analyzed because there was no between-group variability in the present study. Figure 2 displays the mediation model.

Statistical analyses of outcome measures were conducted using Stata/IC version 15.1. The effect of outcome measures in the present study was investigated in different two-level growth models with level-1 modeling the repeated measures within participants, and level-2 modeling the differences of individual growth models across the sample (Kwok et al., 2008). Growth curve models with linear, quadratic and cubic functions were tested by using a likelihood-ratio test to find an acceptable model for the data (Chou et al., 2004). REML was used as the estimation method and an unstructured covariance structure was employed in this analysis. To handle non-normally distributed data and heteroscedasticity in the residuals, we used a robust sandwich estimator to calculate standard errors.

Estimated means and within-group ES were computed for outcome and process measures. Estimated means were calculated by using change trajectories. ES were investigated by subtracting estimated means from observed means at baseline, and finally dividing this number by *SD* of observed values at baseline (Santoft et al., 2019).

To establish the relationship between positive mental health and changes in residual depressive symptoms, correlation analyses were conducted on change scores from baseline to 12-month follow-up.

The statistical significance level was set to *p* = 0.05.

## Results

### Overall Outcomes

A previous publication reported BDI-II and HRSD outcomes (Østergaard et al., 2019), but did not investigate the effect of group-based ACT on positive mental health as measured by MHC-SF. However, the results from the self-reported and clinician-rated measures of depressive symptoms for the sample in this study will also be reported here insofar as they are relevant in interpreting the correlational and mediational analyses central to the purpose of this project.

Participants who received ACT exhibited significant improvement in both self-reported and clinician-rated levels of depression as assessed, respectively, by the BDI-II and HRSD (*ps* < 0.001; see Supplementary Tables S1, S2). The growth curve model also showed a significant increase in positive mental health as assessed by MHC-SF (*p* < 0.001; see Supplementary Table S3). The estimates of time in growth models with quadratic and cubic functions are not interpretable, therefore model-based means of the outcomes are presented in Table 2. The estimated mean values and ES of BDI-II stabilized from 6 to 12 months, while HRSD and MHC-SF continued to improve over 12 months.

**TABLE 2:** Observed baseline values and estimated means and within-group effect sizes during follow-up for outcome and process measures.

	Observed values	Estimated values and effect sizes					
	Baseline	2-months		6-months		12-months	
	*M (SD)*	*M*	*d*	*M*	*d*	*M*	*d*
BDI-II	19.5 (10.85)	15.1	0.41	13.3	0.57	13.3	0.57
HRSD	9.6 (6.25)	6.7	0.56	5.7	0.62	5.5	0.66
MHC-SF	32.0 (14.63)	35.4	0.23	40.1	0.56	40.6	0.59
AAQ-II	27.3 (8.15)	25.2	0.26	22.1	0.64	21.5	0.71
CFQ	30.4 (8.94)	27.5	0.32	23.9	0.73	23.7	0.75
BEVS	11.7 (4.25)	13.0	0.31	14.4	0.64	14.7	0.71
ELS	45.7 (10.46)	48.5	0.27	51.6	0.56	52.5	0.65
PHLMS awareness	30.4 (6.28)	31.8	0.22	33.0	0.41	32.8	0.35
PHLMS acceptance	28.1 (5.75)	29.4	0.22	30.8	0.47	31.1	0.52

*For clarity, all effect sizes are presented as positive values because all measures improved over time.*

The change scores of MHC-SF over 12 months showed a significant negative correlation with similar change scores for both BDI-II, *r* = −0.67, and HRSD, *r* = −0.58 (all *p*s < 0.001).

### Mediation Analyses

The primary purpose of this study was to investigate the mediation of decreases in depressive symptoms and improvement in mental health associated with ACT. In the multilevel mediation model, we first sought to investigate the mediational status of PF as measured by the AAQ-II. Results showed a significant indirect effect of AAQ-II on both self-reported levels of depression (BDI-II) and clinician-rated depression (HRSD) as well as positive mental health (MHC-SF). The relevant path coefficients, indirect effects, and MCIs of the multilevel mediation models for PF are presented in Table 3. Within-group ESs of the AAQ-II were larger than outcome variables at 12-month follow-up and continued to increase over 12 months.

**TABLE 3:** Multilevel coefficients and Monte Carlo confidence intervals for mediation of AAQ-II on respectively BDI-II, HRSD, and MHC-SF.

Outcome	*a*	*b*	*ab*	*c′*	95% MCI
HRSD	−0.482***(0.063)	0.274***(0.041)	−0.132***(0.026)	−0.142**(0.046)	[−0.185, −0.085]
BDI-II	−0.482***(0.063)	0.691***(0.065)	−0.333***(0.054)	−0.130^n.s.^(0.074)	[−0.446, −0.233]
MHC-SF	−0.482***(0.063)	−0.842***(0.085)	0.406***(0.067)	0.310**(0.095)	[0.283, 0.547]

Standard error in parentheses, n.s. = non-significant, ** *p* < 0.005, *** *p* < 0.001.

The mediating effects on PF of the following subprocesses were next investigated: (a) acceptance (measured by PHLMS), (b) awareness (measured by PHLMS), (c) cognitive defusion (measured by CFQ), and (d) values and committed action (measured by BEVS and ELS). Analyses revealed that changes in acceptance, cognitive fusion, values and committed action mediated subsequent increases in PF. There was no significant indirect effect of awareness on PF. The relevant path coefficients, indirect effects, and MCIs of the multilevel mediation models for the subprocesses are presented in Table 4. All the subprocesses, except awareness, continued to increase over 12 months and showed moderate to large ES at 12-month follow-up (range 0.52 –0.76).

**TABLE 4:** Multilevel coefficients and Monte Carlo confidence intervals for mediation of hypothesized mediators on psychological flexibility (AAQ-II).

Mediator	*a*	*B*	*ab*	*c′*	95% MCI
CFQ	−0.534***(0.069)	0.660***(0.038)	−0.353***(0.050)	−0.144**(0.048)	[−0.452, −0.258]
BEVS	0.229***(0.034)	−0.710***(0.102)	−0.162***(0.034)	−0.323***(0.061)	[−0.234, −0.102]
ELS	0.516***(0.084)	−0.396***(0.039)	−0.204***(0.039)	−0.293***(0.057)	[−0.283, −0.132]
PHLMS Awareness	0.156***(0.042)	0.012^n.s.^(0.093)	0.002^n.s.^(0.015)	−0.487***(0.065)	[−0.028, 0.032]
PHLMS Acceptance	0.217***(0.048)	−0.551***(0.066)	−0.112***(0.031)	−0.373***(0.059)	[−0.185, −0.064]

Standard error in parentheses, n.s. = non-significant, ** *p* < 0.005, *** *p* < 0.001.

## Discussion

A multilevel mediation model found that monthly changes in PF mediated reductions in self-reported and clinician-rated levels of depression. These results are consistent with previous findings that have also found an association between PF and depressive symptoms (Forman et al., 2007; Bohlmeijer et al., 2011; Zettle et al., 2011; Fledderus et al., 2013; Pots et al., 2016). Our findings, however, further document the role of PF in reducing depressive symptomatology by including a longer follow-up than previous research. The results also showed that increases in PF mediated increments in positive mental health. Furthermore, we found that ES was larger for PF than for outcomes, suggesting that PF facilitates changes in depression and positive mental health. Collectively, these findings substantiate the conceptual model of human functioning on which ACT is based and underscore the importance of targeting PF to support positive change in depressed individuals. However, it is unclear within such analyses which specific subprocesses, in turn, contribute to increased PF.

We therefore performed secondary mediational analyses on subprocesses of PF to help parse out how it may function as a mechanism of action for therapeutic change. To our knowledge, such a mediational evaluation of the components of PF in relation to it has not previously been undertaken. The results suggest that changes in acceptance, cognitive defusion, values and committed action mediated improvement in PF. Present moment awareness, however, was not found to do so. This finding might be explained by the fact that mindfulness in our group-based ACT primarily focused on being attentive to processes, such as acceptance and cognitive defusion, and less on awareness of different senses. The findings thus correspond with the functional focus of mindfulness in ACT (Hayes et al., 2012a) to not merely increase awareness of sensations, but rather the ability to be more aware and psychologically flexible in the present moment. The results from our analyses support the underlying theory of ACT in suggesting that a number of subprocesses are central to fostering the development of PF and in impacting depression and positive mental health in desired ways. In focusing on increasing awareness of rigid psychological reactions and expanding PF there was limited focus on depression *per se* in group sessions. Rather, more attention was placed on increasing positive mental health and taking steps toward a vital and meaningful life. The focus that group-based ACT in particular places on values and commitment appears to provide an opportunity to enhance the well-being of clients in tailored ways. By broadening the perspective from a limited focus of symptom reduction to also include clarification of what makes life worth living, ACT appears to be a promising approach for altering psychological functioning in multidimensional ways. The degree to which alternative approaches attain similar outcomes through shared or distinct processes is an empirical question that can hopefully be addressed through further research that seeks to identify their key components and associated mechanisms of action.

The ACT model reflects a second order-change strategy, where the form and frequency of depressive thoughts and feelings are not targeted, but rather the context in which they take place (Zettle, 2007). From an ACT perspective, the goal of treatment is not to help participants *feel* better, but rather to help them develop an understanding of themselves and a perspective that facilitates taking valued and meaningful actions. It could be argued that it is therefore important to include a measure that targets life quality, in addition to those assessing depressive symptoms, to provide a comprehensive evaluation of ACT’s impact. In this study, both self-reported and clinician-rated measures of depressive symptoms and a measure of positive mental health changed significantly during a 12-month follow-up. There was a large negative correlation between change scores of both BDI-II and HRSD and MHC-SF, indicating that group-based ACT affected both a reduction in residual depressive symptoms, as well as increases in positive mental health. These results are consistent with those from Fledderus et al. (2012) that ACT promotes well-being in individuals, while also decreasing depressive symptoms. Such overall findings may be especially encouraging if further research is able to determine that enhanced positive mental health functions as a buffer against new depressive episodes.

A criticism of mediational studies in ACT has been that they generally include too few processes of PF. In this project, we included specific measures of the different processes of PF with the exception of self-as-context insofar as, no specific instruments for assessing this process existed at the start of the project. However, recently both the Self-as-Context Scale (Zettle et al., 2018) and the Self Experiences Questionnaire (Yu et al., 2016) have been developed and could be included in future mediational research.

Although our mediational findings indicate that PF and its subprocesses play central roles, they do not establish causality (Kazdin, 2007; Winer et al., 2016). A requirement of establishing mediation is temporal precedence (Murphy et al., 2009). Because of the long intervals between follow-ups, time-lag analyses were not performed. Thus, it was not possible to establish if change in the mediator took place prior to change in other variables of interest. It would have been informative if process and outcome variables had been monitored weekly during the 8 weeks of group-based ACT to see how changes unfold during the intervention and which therapeutic components might be accountable for them. However, to reduce participant burden, this was not undertaken.

Some limitations must be recognized. First, the lack of a control group in our investigation of mediation and positive mental health outcomes prevents us from determining if changes in processes are specific to ACT, and also if improvement in positive mental health was better than no treatment. The generalization of these results must, therefore, be made with care. Second, although it did not affect the results, it must be acknowledged that the inclusion of a small number of participants who fulfilled the formal criteria for current MDD deviated from the preregistered *Clinical Trials* protocol. Third, the majority of participants were female (75%) suggesting that men were underrepresented, especially in light of the 2:1 female-to-male ratio commonly reported for prevalence of depression (Andrade et al., 2003).

A strength of the study was its longitudinal design that made it possible to investigate the relationship between process and outcome measures over 12 months. We included several measures in secondary mediational analyses that cover most of the subprocesses that constitute PF, thereby providing a more extensive evaluation of the role of PF in relapse prevention. The participants were unselected patients in a standard outpatient clinic, thus representing the general population of those seeking mental health services. A further strength of the study involved tracking levels of depression with both clinician-rated and self-reported measurements of depression and levels of positive mental health.

## Conclusion

Findings from the mediational analyses suggest that PF plays an important role in decreasing depressive symptomatology on both clinician-rated and self-report measures and in increasing positive mental health in group-based ACT, and that the subprocesses of acceptance, cognitive defusion, values and committed action contribute to the impact of PF. Group-based ACT not only decreases symptoms of depression, but also increases positive mental health over the course of a 1-year follow up.

## Data Availability Statement

The datasets generated for this study are available on request to the corresponding author.

## Ethics Statement

Approved by the Norwegian Regional Committees for Medical and Health Research Ethics, reference number 2014/1989. We obtained informed, written consent from all participants in the study.

## Author Contributions

TØ contributed to study conception and design, project planning, acquisition of data, analysis and interpretation of data, and drafted the manuscript. TL contributed to study design, project planning, and critical revision. RZ contributed to study conception and design and critical revision. NL contributed to study conception and design, project planning and critical revision. VH contributed to study design, project planning, analysis and interpretation of data, and critical revision. All authors read and approved the final manuscript.

## Conflict of Interest

NL has received consultancy fees and travel expenses from Lundbeck. The remaining authors declare that the research was conducted in the absence of any commercial or financial relationships that could be construed as a potential conflict of interest.
