# Supplementary material for: Psychological Flexibility in Depression Relapse Prevention: Processes of Change and Positive Mental Health in Group-Based ACT for Residual Symptoms
Source: Front Psychol. 2020 Mar 27;11:528. doi: 10.3389/fpsyg.2020.00528 (PMC7119364; doi:10.3389/fpsyg.2020.00528)
Supplement: Supplementary file 1 [file Table_1.DOCX]

Table S1

Growth curve model for estimates of BDI-II

|  |  | Unconditional model | | |  | Conditional model | | |
| --- | --- | --- | --- | --- | --- | --- | --- | --- |
|  |  | Estimate | *SE* | 95 % CI |  | Estimate | *SE* | 95% CI |
| Fixed effects  Intercept  Time  Months, linear  Months, quadratic  Months, cubic |  | 15.58***  -  -  - | 0.95  -  -  - | [13.72, 17.44]  -  -  - |  | 19.58***  -3.14***  0.48**  -0.02* | 1.06  0.75  0.17  0.01 | [17.50, 21.67]  [-4.61, -1.66]  [0.15, 0.81]  [-0.04, 0.00] |
| Random effects  sd (Residuals)  sd (Intercept) |  | 7.32  8.80 | 0.47  0.85 | [6.46, 8.30]  [7.28, 10.64] |  | 6.61  9.28 | 0.57  1.01 | [5.57, 7.83]  [7.50, 11.48] |

Note: *** p < .001, **p<.01, *p<.05
