# Supplementary material for: Psychological Flexibility in Depression Relapse Prevention: Processes of Change and Positive Mental Health in Group-Based ACT for Residual Symptoms
Source: Front Psychol. 2020 Mar 27;11:528. doi: 10.3389/fpsyg.2020.00528 (PMC7119364; doi:10.3389/fpsyg.2020.00528)
Supplement: Supplementary file 2 [file Table_2.DOCX]

Table S2

Growth curve model for estimates of HRSD

|  |  |  | Unconditional model | | |  | Conditional model | | |
| --- | --- | --- | --- | --- | --- | --- | --- | --- | --- |
|  |  |  | Estimate | *SE* | 95 % CI |  | Estimate | *SE* | 95% CI |
| Fixed effects  Intercept  Time  Months, linear  Months, quadratic  Months, cubic |  |  | 7.03***  -  -  - | 0.48  -  -  - | [6.09, 7.97]  -  -  - |  | 9.56***  -1.96***  0.30**  -0.01* | 0.61  0.44  0.11  0.01 | [8.36, 10.55]  [-2.82, -1.10]  [0.10, 0.51]  [-.03, 0.00] |
| Random effects  sd (Residuals)  sd (Intercept) |  |  | 4.39  4.29 | 0.57  0.28 | [3.88, 4.97]  [3.31, 5.58] |  | 3.81  4.65 | 0.33  0.59 | [3.23, 4.51]  [3.64, 5.96] |

Note: *** p < .001, **p<.01, *p<.05
