# Supplementary material for: Psychological Flexibility in Depression Relapse Prevention: Processes of Change and Positive Mental Health in Group-Based ACT for Residual Symptoms
Source: Front Psychol. 2020 Mar 27;11:528. doi: 10.3389/fpsyg.2020.00528 (PMC7119364; doi:10.3389/fpsyg.2020.00528)
Supplement: Supplementary file 3 [file Table_3.DOCX]

Table S3

Growth curve model for estimates of MHC-SF

|  |  | Unconditional model | | |  | Conditional model | | |
| --- | --- | --- | --- | --- | --- | --- | --- | --- |
|  |  | Estimate | *SE* | 95 % CI |  | Estimate | *SE* | 95% CI |
| Fixed effects  Intercept  Time  Months, linear  Months, quadratic |  | 36.68***  -  - | 1.38  -  - | [33.97, 39.38]  -  - |  | 31.65***  2.08***  -0.11*** | 1.42  0.37  0.03 | [28.86, 34.43]  [1.36, 2.80]  [-0.17, -0.05] |
| Random effects  sd (Residuals)  sd (Intercept)  sd (months)  Correlation (months, linear;  intercept) |  | 9.48  13.10  -  - | 8.90  0.90  -  - | [8.32, 10.81]  [11.45, 14.97]  -  - |  | 7.74  13.38  0.68  -0.18 | 0.68  0.97  0.14  0.15 | [6.52, 9.20]  [11.61, 15.42]  [0.46, 1.03]  [-0.46, 0.12] |

Note: Robust standard errors in parentheses, *** p < .001
