# Supplementary material for: Psychological Flexibility in Depression Relapse Prevention: Processes of Change and Positive Mental Health in Group-Based ACT for Residual Symptoms
Source: Front Psychol. 2020 Mar 27;11:528. doi: 10.3389/fpsyg.2020.00528 (PMC7119364; doi:10.3389/fpsyg.2020.00528)
Supplement: Supplementary file 4 [file Table_4.DOCX]

Supplementary Table 4

| Outcome |  | *a* | *b* | *ab* | *c´* | 95% MCI |
| --- | --- | --- | --- | --- | --- | --- |
| HRSD  BDI  MHC-SF |  | -.450^***^(.064)  -.450^***^ (.064)  -.450^***^ (.064) | .246^***^ (.047)  .652^***^ (.073)  -.788^***^ (.095) | -.110^***^ (.026)  -.293^***^ (.053)  .354^***^ (.066) | -.150^**^ (.047)  -.130^n.s.^ (.077)  .247^**^ (.095) | [-.154, -.064]  [-.406, -.195]  [ .234, .494] |

Multilevel coefficients and Monte Carlo confidence intervals for mediation of AAQ-II on respectively BDI-II, HRSD and MHC-SF with 15 participants included in error dropped.

Note: Standard error in parentheses , n.s. = non significant, ^**^ p <.005  ^***^p < .001
