# Supplementary material for: Psychological Flexibility in Depression Relapse Prevention: Processes of Change and Positive Mental Health in Group-Based ACT for Residual Symptoms
Source: Front Psychol. 2020 Mar 27;11:528. doi: 10.3389/fpsyg.2020.00528 (PMC7119364; doi:10.3389/fpsyg.2020.00528)
Supplement: Supplementary file 5 [file Table_5.DOCX]

Table S5

| Mediator |  | *a* | *b* | *ab* | *c´* | 95% MCI |
| --- | --- | --- | --- | --- | --- | --- |
| CFQ  BEVS  ELS  PHLMS Awareness  PHLMS Acceptance |  | -.545^***^(.069)  .203^***^ (.034)  .449^***^ (.087)  .150^***^ (.045)  .232^***^ (.050) | .653^***^ (.041)  -.611^***^ (.113)  -.353^***^ (.042)  .018^n.s.^ (.095)  -.671^***^ (.072) | -.356^***^ (.051)  -.124^***^ (.034)  -.159^***^ (.036)  .003^n.s.^ (.015)  -.155^***^ (.037) | -.109^**^ (.049)  -.323^***^ (.063)  -.305^***^ (.057)  -.449^***^ (.066)  -.303^***^ (.057) | [-.458, -.260]  [-.192, -.069]  [-.236, -.094]  [-.034, .026]  [-.233, -.087] |

Multilevel coefficients and Monte Carlo confidence intervals for mediation of hypothesized mediators on psychological flexibility (AAQ-II) with 15 participants included in error dropped.

Note: Standard error in parentheses, n.s. = non significant, ^**^p <.005, ^***^p < .001
